# Routine care provided by specialists to children and adolescents in the United States (2002-2006)

**DOI:** 10.1186/1472-6963-9-221

**Published:** 2009-12-04

**Authors:** Jose M Valderas, Barbara Starfield, Christopher B Forrest, Luis Rajmil, Martin Roland, Bonnie Sibbald

**Affiliations:** 1 National Primary Care Research and Development Centre, The University of Manchester, UK; 2 Department of Health Policy and Management, Johns Hopkins Bloomberg School of Public Health, Baltimore, MD, USA; 3 Department of Pediatrics, Children's Hospital of Philadelphia, University of Pennsylvania School of Medicine, Philadelphia, PA, USA; 4 Catalan Agency for Health Technology Assessment and Research (CAHTA), Carrer de Roc Boronat,81-95 (2a planta), 08005, Barcelona, Spain; 5 Health Services Research Unit, IMIM-Hospital del Mar, Carrer del Dr. Aiguader, 08003, Barcelona, Spain; 6 School of Clinical Medicine, General Practice and Primary Care Research Unit, University of Cambridge, UK

## Abstract

**Background:**

Specialist physicians provide a large share of outpatient health care for children and adolescents in the United States, but little is known about the nature and content of these services in the ambulatory setting. Our objective was to quantify and characterize routine and co-managed pediatric healthcare as provided by specialists in community settings.

**Methods:**

Nationally representative data were obtained from the National Ambulatory Medical Care Survey for the years 2002-2006. We included office based physicians (excluding family physicians, general internists and general pediatricians), and a representative sample of their patients aged 18 or less. Visits were classified into mutually exclusive categories based on the major reason for the visit, previous knowledge of the health problem, and whether the visit was the result of a referral. Primary diagnoses were classified using Expanded Diagnostic Clusters. Physician report of sharing care for the patient with another physician and frequency of reappointments were also collected.

**Results:**

Overall, 41.3% out of about 174 million visits were for routine follow up and preventive care of patients already known to the specialist. Psychiatry, immunology and allergy, and dermatology accounted for 54.5% of all routine and preventive care visits. Attention deficit disorder, allergic rhinitis and disorders of the sebaceous glands accounted for about a third of these visits. Overall, 73.2% of all visits resulted in a return appointment with the same physician, in half of all cases as a result of a routine or preventive care visit.

**Conclusion:**

Ambulatory office-based pediatric care provided by specialists includes a large share of non referred routine and preventive care for common problems for patients already known to the physician. It is likely that many of these services could be managed in primary care settings, lessening demand for specialists and improving coordination of care.

## Background

Specialist physicians provide an increasing share of health care for children and adolescents [1], at least to some extent associated with the increasing number of pediatric patients with chronic conditions [2,3]. Little is known about who is providing specialized care for these patients and what is the content of this care in the community [4-6], where most of the health care for children and adolescents is provided [7].

The current debate on the future needs of health care workforce needs to be informed not only by trends in absolute and relative numbers of physicians, but also by the content and the type of the care provided by them [8-12]. Information on care as provided by specialists is also fundamental to the organization and coordination of services that fulfill the principles of the Medical Home model [13-15]. The recent publication of a report on adolescent health services by the National Research Council and the Institute of Medicine that recommends the development of new systems for providing services that are accessible, acceptable, appropriate, effective, and equitable, makes empirical information particularly timely [16].

In order to improve the understanding of what US specialists do in caring for children and adolescents patients in their area of special interest we aimed to describe the nature and content of the specialist care focusing on care provided in the community. In particular, we focused our analysis on the burden of routine follow-up and preventive care as well as the degree to which care is shared with primary care physicians.

## Methods

Data were obtained from the National Ambulatory Medical Care Survey (NAMCS), United States, for five consecutive years (2002 to 2006).

The survey included visits made to non-federally employed, office-based physicians in the United States. A multistage probability design was used with probability samples of 112 geographic sampling units, physician practices within geographic units, and patient visits within practices. Non-federally employed physicians (excluding those in the specialties of anesthesiology, radiology, and pathology) who are classified by the American Medical Association (AMA) or the American Osteopathic Association (AOA) as primarily engaged in office-based patient care were randomly selected. Selected physicians completed questionnaires for a systematic random sample of all patient visits made during 1 week (yearly response rates ranging from: 70.4% (2002) to 58.9% (2006)). Additional details of the survey's methods are available elsewhere [17-19]. In this study, we included only visits for patients aged 18 or younger.

### Physician specialty

The principal specialty of a physician was self-designated at the time of the survey [17]. Because our focus was on specialists care, we excluded all family and general practitioners, general internists and general paediatricians [20,21]. Physician specialty was then classified into 3 mutually exclusive groups[20,22]: medical specialists, surgical specialists, and psychiatrists.

### Primary diagnosis

Primary diagnosis for each visit was recorded as free text by the physician and was coded according to the International Classification of Diseases, 9th Revision, Clinical Modification (ICD-9CM)[23]. More than 10.000 different ICD-9CM codes were aggregated into 264 Expanded Diagnostic Clusters (EDC). These clusters are clinically homogeneous groups of diagnostic codes that were developed by Johns Hopkins University[24,25].

### Type of visit

A classification of types of visits was developed based on two major visit attributes as reported by the specialist: "Previous knowledge of the health problem" (5 categories: "New patient", "Known patient, new problem", "Known patient, known problem (recurrence)", "Known patient, known problem (routine)", and "Other")(Table 1), and "Visit orientation" (3 categories: "Primary care", "Referred specialty care", and "Non referred specialty care")[19].

**Table 1:** Classification of visit type: Previous knowledge of the health problem.

Major reason for this visit*	Known patient?	
	No	Yes
Acute problem	*New patient*	*Known patient, new problem*
Pre- or Post-surgery	*New patient*	*Known patient, new problem*
Chronic problem, flare-up	*New patient*	*Known patient, known problem (recurrence)*
Chronic problem, routine	*New patient*	*Known patient, known problem (routine or preventive)*
Preventive care	*New patient*	*Known patient, known problem (routine or preventive)*

E.g.: A visit for an acute problem by a known patient would be categorized as *Known patient, new problem*. These categories were applied to both primary care non primary care visits, but the focus of the subsequent analyses is restricted to non primary care visits only.

### Shared care and reappointments

Since specialized health care is expected to be coordinated with that provided by the patient's primary care physician[14,26], we measured shared care reported by the specialist as a proxy for coordination. Shared care has been defined as the joint participation of primary care physicians and specialist care physicians in the planned delivery of care[27]. Physicians were instructed to report that they shared a patient's care if they were providing care for a portion of the patient's total treatment for his/her condition AND other physician(s) were also providing care[24](this information was not included in the 2005 and 2006 surveys).

Participating physicians also reported whether a subsequent follow-up appointment had been suggested to the patient at the end of the visit (reappointment).

### Case-mix

The Johns Hopkins Adjusted Clinical Groups (ACG) Case-Mix System http://www.acg.jhsph.edu was used for case-mix adjustment [24,28]. The system determined whether the condition identified as the primary diagnosis was related to very high expected annual resource use (Major ADG) or not.

### Data analysis

The unit of analysis was the visit throughout. Sampling weights that accounted for the multistage sampling design were obtained from the National Center for Health Statistics. These weights were used to obtain national estimates of the overall numbers of visits and descriptives for all the variables[17,18]. Likelihood ratios accounting for the multistage sampling design were used in testing all statistical associations between categorical variables[29]. In the bivariate comparison of types of specialists, physician specialty was the independent variable and patient and visit variables (including type of visit) were the dependent variables. Associations between physician specialty and continuous dependent variables were tested by means of the overlap of confidence intervals for the estimates of each specialty group.

Two multivariable logistic regression models were constructed for the comparisons between referrals and non referrals among visits to specialists adjusting for patient's age, sex, ethnicity, insurance, morbidity burden, and physician specialty (corresponding categories are listed in Table 2) [30]. In these analyses, the occurrence of a referral was the independent variable and shared care and reappointment were the dependent variables.

**Table 2:** Descriptives of selected variables for all visits.

			Medical Specialists	Surgical Specialists	Psychiatrists	Generalists	Total
**All visits**		N	64,238,724	97,887,455	24,399,804	739,160,674	925,686,657
		%	6.9%	10.6%	2.6%	79.8%	100.0%

**Sex**	Female	N	30,296,129	52,765,703	8,968,052	356,775,047	448,804,931
		%	47.2%	53.9%	36.8%	48.3%	48.5%
	Male	N	33,942,595	45,121,752	15,431,752	382,385,627	476,881,726
		%	52.8%	46.1%	63.2%	51.7%	51.5%

**Age**		Mean	10.4	10.8	12.7	6.3	7.3
		95%CI	[9.8-11.0]	[10.3-11.2]	[12.3-13.0]	[6.2-6.5]	[7.1-7.4]

Ethnicity*	White, non Hispanic	N	47,841,774	70,737,424	19,393,170	508,728,742	646,701,110
		%	74.5%	72.3%	79.5%	68.8%	69.9%
	Black, non Hispanic	N	6,148,335	9,938,910	2,203,970	73,553,754	91,844,969
		%	9.6%	10.2%	9.0%	10.0%	9.9%
	Hispanic	N	8,095,064	14,783,223	2,326,799	123,906,567	149,111,653
		%	12.6%	15.1%	9.5%	16.8%	16.1%
	Asian/Pacific Islander	N	2,153,551	2,427,898	475,865	32,971,611	38,028,925
		%	3.4%	2.5%	2.0%	4.5%	4.1%

**Insurance**	Private insurance	N	47,015,087	65,992,342	11,944,789	495,028,949	619,981,167
		%	76.7%	70.3%	50.9%	69.6%	69.7%
	Medicare	N	870,996	1,120,416	266,621	9,902,733	12,160,766
		%	1.4%	1.2%	1.1%	1.4%	1.4%
	Medicaid/SCHIP	N	10,570,492	24,127,052	6,941,932	187,878,504	229,517,980
		%	17.2%	25.7%	29.6%	26.4%	25.8%
	No insurance	N	2,870,618	2,622,247	4,317,028	18,190,300	28,000,193
		%	4.7%	2.8%	18.4%	2.6%	3.2%

**Past visits**	0	N	3,834,448	6,450,511	226,267	40,817,779	51,329,005
		%	7.82%	9.50%	1.03%	6.07%	6.32%
	1-2	N	20,090,835	31,721,992	4,067,295	227,905,155	283,785,277
		%	40.99%	46.74%	18.48%	33.88%	34.96%
	3-5	N	14,078,175	18,858,710	6,643,475	218,838,397	258,418,757
		%	28.72%	27.78%	30.19%	32.53%	31.84%
	6 or more	N	11,010,175	10,842,581	11,071,313	185,215,197	218,139,266
		%	22.46%	15.97%	50.31%	27.53%	26.88%

**Time spent**		Mean	18.0	17.8	32.6	15.5	16.38
**with the physician**		95%CI	[15.9-20.4]	[15.9-19.7]	[29.9-35.4]	[15.1-15.9]	[15.9-16.8]

**Major**	Yes	N	54,58,393	8,055,130	8,814,148	1,995,243,756	44,436,728
**ADG as**		%	8.5%	10.2%	36.1%	2.7%	4.8%
**Primary**	No	N	58,757,994	87,941,942	15,601,774	719,026,731	881,328,441
**Diagnosis**		%	91.5%	89.8%	63.9%	97.3%	95.2%

**Number of**		Mean	1.7	1.4	1.7	1.3	1.2
**diagnoses**		95%CI	[1.5-1.9]	[1.4-1.5]	[1.5-1.8]	[1.2-1.3]	[1.2-1.3]

**Visit**	Primary care	N	5,166,752	6,091,280	1,299,516	644,742,251	657,299,799
**orientation**		%	8.0%	6.2%	5.3%	87.2%	71.0%
	Specialty care	N	59,417,648	92,155,172	23,219,654	94,641,064	268,386,858
		%	92.0%	93.8%	94.7%	12.8%	29.0%

CI 95%: Confidence Interval; ADG: Aggregated Diagnostic Groups

^† ^All differences across specialists (generalists vs medical specialists vs surgical specialists vs psychiatrists) were statistically significant at the defined α level (0.05) for all variables.

The statistical software used included SPSS, version 14.0, with instructions for use of the Complex Samples module as recommended by the National Center for Health Statistics[31], and the Johns Hopkins ACG Case-Mix System, version 8.1.

## Results

### Description of visits

Overall, 20.2% of the available estimated 925,686,657 visits (28,571 non weighted visits) were to specialists (Table 2, results for generalists included for illustrative purposes only). In one in four visits, the physician reported having seen the patient at least 6 times in the previous 12 months. This was the case in 50% of all visits to psychiatrists. Significant differences were observed by physician specialty group in age, sex, and insurance status of patients. Visits for medical specialists were similar in proportion of visits for female patients (47.2% vs. 53.9%), mean age (10.4 years vs. 10.8 years). The most frequent type of insurance in all visits was private insurance followed by Medicaid/SCHIP for all specialties, but there were significant differences between specialty groups. The proportion of visits for patients with private insurance was highest for medical specialists (76.7%) while Medicaid/SCHIP was highest among psychiatrists (29.6). Visits for psychiatrists were significantly longer than those for other specialties and there were also statistically significant differences in case-mix and mean number of diagnoses across the defined physician specialty groups.

Subsequent analyses are restricted to visits to specialists and where the focus of the visit was specialty care (173.968.435 estimated visits).

### Routine follow-up in visits to specialists

Routine or preventive visits by known patients emerged as the most frequent type of visit, accounting for 71,896,865 estimated visits overall (41.3%), and for the great majority of all visits to medical specialists (51.2%) and psychiatrists (73.8%) (Table 3). Visits for a new problem by a known patient were the most frequent among visits for surgical specialists (34.9%). Visits for new patients ranged from 1 in 10 for psychiatrists to 1 in 4 for medical specialists and 1 in 3 for surgical specialists. Only 2.2% of visits were in the category "Other".

**Table 3:** Type visits and frequency of reappointments by physician specialty group (specialty care visits only).

		Medical Specialists			Surgical Specialists			Psychiatrists			All visits		
		Referred	Non referred	*Overall*	Referred	Non referred	*Overall*	Referred	Non referred	*Overall*	Referred	Non referred	Overall
**New patient**	N	7,476,112	7,295,856	*14,771,968*	18,745,793	9,877,375	*28,623,168*	1,250,196	944,852	*2,195,048*	27,472,101	18,118,083	*45,590,184*
	%	40.0	18.1	*25.0*	51.2	17.9	*31.2*	23.6	5.3	*9.5*	45.3	16.0	*26.2*
**Known patient, new problem**	N	1,674,553	4,895,022	*6,569,575*	10,121,704	21,879,415	*32,001,119*	317,813	554,923	*872,736*	12,114,070	27,329,360	*39,443,430*
	%	9.0	12.1	*11.1*	27.6	39.7	*34.9*	6.0	3.1	*3.8*	20.0	24.1	*22.7*
**Known patient, known** **Problem: recurrence**	N	1,623,935	4,142,757	*5,766,692*	1,748,531	3,085,111	*4,833,642*	351,669	2,219,454	*2,571,123*	3,724,135	9,447,322	*13,171,457*
	%	8.7	10.3	*9.8*	4.8	5.6	*5.3*	6.6	12.5	*11.1*	6.1	8.3	*7.6*
**Known patient, known problem: routine or preventive**	N	7,644,382	22,611,827	*30,256,209*	5,605,670	18,996,051	*24,601,721*	3,279,692	13,759,243	*17,038,935*	16,529,744	55,367,121	*71,896,865*
	%	40.9	56.0	*51.2*	15.3	34.4	*26.8*	61.8	77.3	*73.8*	27.3	48.8	*41.3*
**Other**	N	261,137	1,446,391	*1,707,528*	398,573	1,337,952	*1,736,525*	106,193	316,253	*422,446*	765,903	3,100,596	*3,866,499*
	%	1.4	3.6	*2.9*	1.1	2.4	*1.9*	2.0	1.8	*1.8*	1.3	2.7	*2.2*

Shared care*	N	5,513,663	4,828,106	*10,341,769*	8,613,939	6,378,177	*14,992,116*	623,457	1,015,185	*163,8642*	14,751,059	12,221,468	*26,972,527*
	%	44.1	16.2	*24.4*	37.5	18.2	*26.2*	16.4	9.0	*10.9*	37.6	16.2	*23.5*

**Reappointments**	N	14,071,536	28,907,553	*42,979,089*	24,695,947	38,267,967	*62,963,914*	4,629,558	16,782,829	*21,412,387*	43,397,041	83,958,349	*127,355,390*
	%	75.3	71.6	*72.8*	67.4	69.4	*68.6*	87.3	94.7	*92.7*	*71.6*	*74.1*	*73.2*

**Overall**	N	18,680,119	40,391,853	*59,071,972*	36,620,271	55,175,904	*91,796,175*	5,305,563	17,794,725	*23,100,288*	60,605,953	113,362,482	173,968,435
	%	100	100	*100*	100	100	*100*	100	100	*100*	100	100	*100*

* All overall differences between specialties statistically significant at the defined α level (0.05). All differences for referred and non referred visits statistically significant for all types of visits, shared care and reappointments, except for reappointments for medical specialist and psychiatrists.

*Information on shared care only available for 114.641.238 visits (65.6%)

Three specialties accounted for about well over half of all routine and preventive care visits: psychiatry (25%), allergy and immunology (17.8%), dermatology (11.7%). Comparatively, visits to these specialists accounted only for 34.9% of all visits when all visit types were considered. Seven diagnostic groups accounted as well for slightly more than half of all routine and preventive visits (52.7%) (Table 4).

**Table 4:** Expanded Diagnostic Clusters accounting for at least 50% of all routine visits to specialists for specialized care.

Expanded diagnostic clusters	Percentage (routine/preventive visits) (standard error)	Percentage (all visits) (standard error)
Attention deficit disorder	10.8 (1.3)	5.2 (0.5)
Allergic rhinitis	9.8 (2.7)	5.2 (1.2)
Sebaceous glands disorders	7.5 (0.8)	5.3 (0.5)
Schizophrenia and affective psychoses	7.1 (1.3)	3.9 (0.6)
Anxiety and neuroses	6.0 (0.8)	3.4 (0.4)
Pregnancy and labour	5.6 (0.9)	2.3 (0.3)
Asthma	5.2 (1.2)	3.4 (0.9)

### Referred and non referred care

The estimated 60,605,953 visits for patients referred from other physicians constituted a third of all specialty care visits (Table 3). This proportion was highest for 39.9% for surgical specialists, followed by medical specialists (31.6%) and was lowest for psychiatrists (23.0%). Although there were no differences in gender, ethnicity and morbidity burden between referred and non referred visits, patients were significantly younger in referred visits (9.8 years vs 11.3 years, p < 0.05). Referred visits were longer than non referred visits (22.8 min vs 18.5 min, p < 0.05) also included a significantly slightly higher proportion of patients with private insurance (69.6% vs 66.3%, p < 0.05).

The most frequent reason for visit in referred care visits was a new patient (45.3%), while non referred care was most frequently for routine or preventive care (48.8%). Routine or preventive care for non referred patients was the most common type of visit, accounting for almost as many visits as all other categories of referred visits combined (55,367,121 vs 60,605,953).

### Shared care and reappointments

Specialists reported sharing care for the patient with another physician in only 23.5% of all visits. Shared care was more likely to be reported in visits for referred patients than for non-referred patients (37.6% vs 16.2%, p < 0.05; adjusted OR = 2.90; IC95%:2.19-3.84).

A reappointment was scheduled in about 3 out of 4 visits. Overall, every 1 out of 2 reappointments resulted from a routine or preventive care visit (47.4%). The likelihood of a reappointment in referred visits was similar to that for non referred visits (71.6% vs 74.1%, p = 0.20; adjusted OR = 0.93; IC95%:0.76-1.14).

## Discussion

Our analysis of about 174 million ambulatory visits to office based specialists by children and adolescents in 2002-2006 demonstrated clear patterns in the provision of health care. Routine and preventive care for patients already known to the physician accounted for more than 40% of visits. About three quarters of visits result in a reappointment with the same specialist regardless of whether the patient had been referred for that visit or not. Referrals by other professionals accounted for about a third of all specialty care visits. For patients without a referral, specialists were about three times less likely to share the patient's care with another physician.

### Limitations of the study

First, all information included in the study was based on physician report. Available evidence regarding the validity of NAMCS data suggests that only self-report of visit duration seems particularly susceptible to over-estimation[32], but we cannot completely rule out other biases. It is difficult to predict how they may have eventually affected our observations in our study.

Second, the distinction between referred and non referred patients could in part be an artefact of follow-up [33-35]. For patients already known to the physician, specialists might not have considered the visit to be a referral even if it was. Nonetheless, physicians were instructed at the time of data collection that referral had to have occurred in relation to that particular visit[24].

Third, physicians reported sharing care with another physician in about a third of all referred care visits. This low percentage might signal a different understanding in the distribution of responsibilities between the referring physician and the specialist [36,37].

Fourth, the unit of analysis was the visit rather than the patient, thus limiting our ability to make any inference about the patients. However, this does not modify our conclusions focusing on physicians activity, better quantified in terms of visits than patients.

### Policy implications

We have shown that routine and preventive visits are common among specialists in office based practice, as well as the source of future such visits through follow-up appointments. This was especially true for non referred care. The low level of shared care and the high levels of routine follow up suggest that primary care physicians are not being incorporated into follow up of referred problems. If they were, it could lower the demand on specialists.

There is little evidence to suggest how frequently patients with common chronic conditions need specialist follow up, and there is considerable variation in the frequencies and intervals at which specialists request their patients to make return visits. In countries with well developed systems of primary care, the routine follow up of patients with common chronic conditions is undertaken in primary care[38,39].

The results of our study suggest that some of the activity performed by specialists working in a specialist role in the community could also be done in primary care. Primary care professionals are accountable for the large majority of personal health care needs[40], and much routine follow up and preventive activities now carried out by specialists could be transferred to the primary care setting. The superiority of care as provided by specialists for adults for common conditions has been recently called into question[41], and current available evidence does not favor one type of professionals over the other in the care of children and adolescents[5].

Greater efficiency might be achieved by having the primary care practitioner do the follow-up care, allowing specialists to focus on those aspects of care which demand their unique skills[36,42]. Interventions that promote efficient contact betweenproviders would need needed in order to ensure an effective communication between primary care physicians and specialists[37,43]. This alternative would be also consistent with the concept of a Medical Home and might be feasible for a substantial proportion of the about 55 million yearly office based ambulatory visits to specialists related to non referred routine management of known patients [12,14].

Inexorable increases in costs of care, the imperative of continuity of care, and the emergence of the Medical Home model appear to be sufficient justification for re-assessing the appropriate relative roles of primary care and specialist physicians.

Our data demonstrate that a handful of conditions account for about half of all ambulatory visits to specialists in children and adolescents in the US. More intensive training of generalists on the management of these particular clinical areas would result in an increased offer for these services within primary care and in increased continuity of care. The same principle would apply to other countries and health systems, should these observations be confirmed in similar studies.

## Conclusion

Ambulatory office-based pediatric care provided by specialists includes a large share of non referred routine and preventive care for common problems for patients already known to the physician. It is likely that many of these services could be managed in primary care settings, lessening demand for specialists and improving coordination of care.

## Lists Of Abbreviations

ACG: Johns Hopkins Adjusted Clinical Groups; ADG: Aggregated Diagnostic Groups; EDC: Expanded Diagnostic Clusters; NAMCS: National Ambulatory Medical Care Survey; US: United States.

## Competing interests

The authors declare that they have no competing interests.

## Authors' contributions

JMV, CBF and BS designed the study. JMV accessed the data and performed the statistical analysis. All authors helped with the interpretation of the data. JMV and BS drafted the first version of the manuscript, and all authors contributed to subsequent versions and revised it critically for important intellectual content. All authors read and approved the final manuscript.

## Pre-publication history

The pre-publication history for this paper can be accessed here:

http://www.biomedcentral.com/1472-6963/9/221/prepub
